# Ghrelin Receptor Influence on Cocaine Reward is Not Directly Dependent on Peripheral Acyl-Ghrelin

**DOI:** 10.1038/s41598-019-38549-z

**Published:** 2019-02-12

**Authors:** Cody J. Wenthur, Ritika Gautam, Bin Zhou, Leandro F. Vendruscolo, Lorenzo Leggio, Kim D. Janda

**Affiliations:** 1 Department of Chemistry, The Scripps Research Institute, La Jolla, CA USA; 2 Neurobiology of Addiction Section, National Institute on Drug Abuse Intramural Research Program, National Institutes of Health, Baltimore, MD USA; 3 Section on Clinical Psychoneuroendocrinology and Neuropsychopharmacology, National Institute on Alcohol Abuse and Alcoholism Division of Intramural Clinical and Biological Research and National Institute on Drug Abuse Intramural Research Program, National Institutes of Health, Bethesda, MD USA; 4 Center for Alcohol and Addiction Studies, Department of Behavioral and Social Sciences, Brown University, Providence, RI USA; 5 Department of Immunology and Microbial Science, The Skaggs Institute for Chemical Biology, and The Worm Institute for Research and Medicine (WIRM), The Scripps Research Institute, La Jolla, CA USA; 6 Present Address: Department of Pharmacy, University of Wisconsin – Madison, Madison, WI USA

## Abstract

The peptide hormone acyl-ghrelin and its receptor, GHSR_1a_, represent intriguing therapeutic targets due to their actions in metabolic homeostasis and reward activity. However, this pleotropic activity makes it difficult to intervene in this system without inducing unwanted effects. Thus, it is desirable to identify passive and active regulatory mechanisms that allow differentiation between functional domains. Anatomical restriction by the blood brain barrier represents one major passive regulatory mechanism. However, it is likely that the ghrelin system is subject to additional passive mechanisms that promote independent regulation of orexigenic behavior and reward processing. By applying acyl-ghrelin sequestering antibodies, it was determined that peripheral sequestration of acyl-ghrelin is sufficient to blunt weight gain, but not cocaine rewarding effects. However, both weight gain and reward-associated behaviors were shown to be blocked by direct antagonism of GHSR_1a_. Overall, these data indicate that GHSR_1a_ effects on reward are independent from peripheral acyl-ghrelin binding, whereas centrally-mediated alteration of energy storage requires peripheral acyl-ghrelin binding. This demonstration of variable ligand-dependence amongst functionally-distinct GHSR_1a_ populations is used to generate a regulatory model for functional manipulation of specific effects when attempting to therapeutically target the ghrelin system.

## Introduction

**Figure 1:**
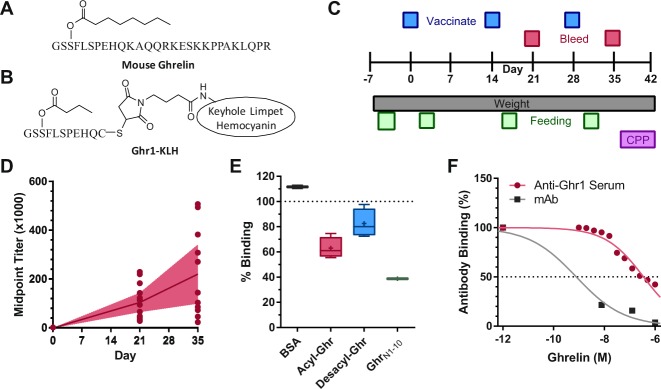
Antibody Generation During Vaccination Against Ghrelin. (**A**) Structure of acyl ghrelin peptide. (**B**) Structure of the Ghr1 hapten with GMBS-mediated linkage to KLH. (**C**) Study timeline showing interventions and outcome measurements. (**D**) Midpoint titers following administration of the Ghr1 vaccine (Day 21, *n* = 14; day 35, *n* = 11). (**E**) Antibody binding to Ghr1-BSA coated chip in the presence of a 10 µM concentration of BSA (*n* = 2), GhrN1-10 (*n* = 2), acyl-ghrelin (*n* = 4), or desacyl-ghrelin (*n* = 4). (**F**) Antibody binding to Ghr1-BSA coated chip from vaccinated mouse serum or from monoclonal anti-ghrelin antibodies in the presence of acyl-ghrelin.

The hormone acyl-ghrelin is a 28-amino acid peptide containing an octanoic ester linkage on Ser^3^, a thus-far unique post-translational modification within mammalian systems (Fig. 1A)^1,2^. To generate the active form of the hormone, preproghrelin is synthesized, processed, and then secreted by endocrine cells located primarily in the stomach, with activation by ghrelin-O-acyltransferase (GOAT) occurring during processing^3,4^. The acyl-ghrelin acts on growth hormone secretagogue receptor 1a (GHSR_1a_), altering metabolic parameters such as energy storage and glucose-homeostasis^5–7^. In addition, acyl-ghrelin has been identified as a key regulator of appetite, acting as an orexigenic signal and leading to feelings of hunger^8,9^. Interestingly, ghrelin signaling has also more recently been implicated in alteration of learning, memory, and reward processing^10–13^. In light of this pleotropic activity, the acyl-ghrelin signaling system has variously been promoted as a point of intervention for diabetes, obesity, anorexia, cachexia, and alcohol and substance use disorders, among others^14,15^.

However, because this signaling system regulates multiple physiologic outcomes, a key goal moving forward is to identify mechanisms in place that will allow an outcome of interest to be suitably modified without introducing unwanted or adverse effects due to global perturbation of ghrelin signaling^16^. One inherent mechanism that has been identified for differentially regulating activity amongst different populations of GHSR_1a_ in rodents is the limited ability of acyl-ghrelin to penetrate the blood-brain barrier (BBB)^17,18^. Considering acyl-ghrelin’s peripheral site of synthesis, it is not surprising that some actions of acyl-ghrelin are exclusively regulated by GHSR_1a_ signaling in the periphery^2,16^. Nevertheless, in cases such as glucose homeostasis, acyl-ghrelin acts through a combination of central and peripheral signals^7,19,20^. Indeed, the most well-studied acyl-ghrelin functions, including orexigenic and rewarding effects, appear to be mostly reliant on GHSR_1a_ signaling in the central nervous system (CNS), where acyl-ghrelin access is more limited and concentrations are naturally much lower^21–25^.

Considering the locally restricted access of peripheral acyl-ghrelin to many regions within the CNS, acyl-ghrelin’s central effects on appetite have been proposed to be modified by remote signaling through vagal afferents^16,26,27^. However, recent results indicate that hypothalamic neurons alone are required for orexigenic activity^28–30^. Direct activation of GHSR_1a_ in the arcuate nucleus of the hypothalamus (ARC) occurs through peripheral hormone access *via* the cerebrospinal fluid (CSF)^16,31^. It has also been proposed that this direct hypothalamic activation may potentially be supported through central synthesis of acyl-ghrelin, though this remains controversial^3,8,30,32,33^. Intriguingly, acyl-ghrelin’s support of homeostatic feeding through the ARC has been demonstrated to be functionally separable from its role in food reward^22,34^. Acyl-ghrelin access to ARC is thought to result in indirect neuronal communication with deeper brain structures as a means to modulate motivated responses for food^16,35,36^. Regardless, none of the mechanisms invoked to explain homeostatic or hedonic feeding behavior are sufficient to account for the ‘enigmatic’ GHSR_1a_ expression in mesolimbic CNS structures^37–39^. Indeed, GHSR_1a_ expression is relatively high in the mesolimbic dopaminergic system involved with reward function, where there is little support for either peripheral access or central synthesis of acyl-ghrelin *in situ*^10,22,40–43^. These mesolimbic dopaminergic pathways do retain their ability to functionally respond to exogenously applied peripheral acyl-ghrelin, such as inducing conditioned place preference (CPP), or modifying CPP resulting from cocaine, although indirect activation through the hypothalamus remains a likely mechanism for these rewarding effects^16,44,45^. However, it is well established in *in vitro* studies that GHSR_1a_ has high levels of intrinsic activity in the absence of ligand binding^44,46–51^. Furthermore, it has been shown that GHSR_1a_ can alter neuronal signaling in the absence of acyl-ghrelin binding simply by forming heterodimeric constructs with dopamine receptors^52–56^. These observations support the hypothesis that under normal physiologic conditions, hypothalamic GHSR_1a_ control of weight gain and feeding are regulated by acyl-ghrelin binding, whereas mesolimbic GHSR_1a_ control of reward signaling occurs via a mechanism that is independent from direct acyl-ghrelin binding.

Widespread interest in altering ghrelin signaling has led to the development of multiple independent strategies to modulate the acyl-ghrelin—GHSR_1a_ signaling system. Although genetic ablation of ghrelin producing cells and GHSR_1a_ with germline deletions had effects across multiple physiologic systems, both germline and inducible ablation failed to reduce appetite or induce weight loss^57–63^. Non-genetic strategies to either directly block GHSR_1a_ using small molecules or neutralize acyl-ghrelin through administration of acyl-ghrelin-binding antibodies have demonstrated reductions in feeding and limited weight gain^64–70^. One possible reason for different outcomes between genetic and non-genetic interventions is that the window to observe orexigenic response to loss of ghrelin signaling may be temporally limited due to compensatory adaptations by redundant or overlapping signals^71^. Importantly, the non-genetic interventions themselves have different conditions in which they prevent GHSR_1a_ signaling; the small molecule approach blocks any ligand-dependent, receptor-associated signals, whereas the antibody approach blocks only those signals reliant on ghrelin binding. Therefore, these interventions present an interesting opportunity to identify whether GHSR_1a_-mediated weight gain, feeding, and reward are mediated by different mechanisms. If the acyl-ghrelin binding-independent hypothesis of brain reward function is correct, it should be possible to induce orexigenic alterations without influencing reward processing by modulating acyl-ghrelin access to central GHSR_1a_ using acyl-ghrelin antibodies. Conversely, blockade of ghrelin-mediated reward signaling should be possible following direct antagonism of GHSR_1a_, but not through perturbation of acyl-ghrelin penetration into the CNS.

## Results

### Vaccine Induction of Anti-Ghrelin Antibodies Alters Weight Gain, but not Reward

The hapten Ghr1-KLH, designed to reflect the N-terminal portion of acyl-ghrelin, was selected to generate functional anti-acyl -ghrelin antibodies (Fig. 1B). Synthesis resulted in the generation of bioconjugates presenting 5–13 copies of the hapten (Supplemental Table 1). This hapten was administered to mice alongside the adjuvants alum and CpG oligodeoxynucleotide (ODN) 1826 as part of a vaccination design where antibody production, weight change, feeding behavior, and cocaine reward were measured over time (Fig. 1C). Treatment with this active vaccine led to the production of anti-acyl-ghrelin antibodies, with increasing titers observed following each boost (Fig. 1D). The polyclonal antibody pool produced using this method was shown to bind the Ghr1 hapten and full length acyl-ghrelin, with less recognition of desacyl-ghrelin and no cross-reactivity with BSA, as measured by surface plasmon resonance (SPR) (Fig. 1E). The affinity of the polyclonal antibodies was relatively modest (349 nM) compared to the sub-nanomolar affinity of previously isolated monoclonal antibodies targeting this region of acyl-ghrelin (Fig. 1F)^69^.

**Figure 2:**
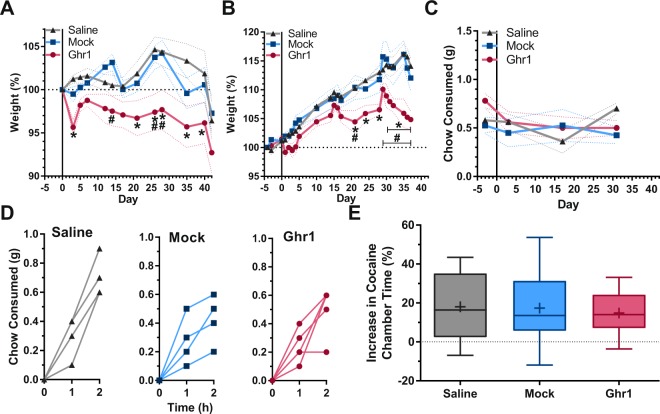
Effects of Vaccination Against Ghrelin on Weight, Feeding, and Reward in Mice. (**A**) Normalized body weight over time in 12–14-week- old, male Swiss Webster mice following treatment with saline (*n* = 8), or vaccination with KLH (*n* = 9), or Ghr1-KLH (*n* = 9) [Repeated measures two-way ANOVA (*P*_interaction_ = 0.0237, F_22, 276_ = 1.70; ^#^<0.05 vs. Mock; *<0.05 vs Saline, Bonferroni correction)]. (**B**) Normalized body weight over time in 6–8 -week -old, male Swiss Webster mice following treatment with saline (*n* = 5), vaccination with KLH (*n* = 4), or Ghr1-KLH (*n* = 5) [Repeated measures two-way ANOVA (*P*_*i*nteraction_ < 0.0001, F_44, 242_ = 2.88; ^#^<0.05 vs. Mock; *<0.05 vs Saline, Bonferroni correction)]. (**C**)Total chow consumption following treatment with saline (*n* = 5), KLH (*n* = 4), or Ghr1-KLH (*n* = 5) [Repeated measures two-way ANOVA (*P*_interaction_ = 0.0900, F_6, 33_ = 2.03). (**D**) Chow consumption on day 31 following treatment with saline (*n* = 5), KLH (*n* = 4), or Ghr1-KLH (*n* = 5) [Repeated measures two-way ANOVA (*P*_interaction_ = 0.0709, F_4, 22_ = 2.51). (**E**) Conditioned place preference for cocaine (20 mg/kg) in mice treated with saline (*n* = 10), KLH (*n* = 17), or Ghr1-KLH (*n* = 13), [One-Way ANOVA (*P* = 0.6536 F_2, 37_ = 0.43)] Data shown as mean ± SEM (a–c), individual data points (d), or median with quartiles ±5–95% CI; +, mean (e).

Nevertheless, the Ghr1-KLH vaccine induced significant weight loss compared to the saline and mock vaccine control groups over the five-week study in 12–14-week-old, male Swiss Webster mice (Fig. 2A). The vaccine also blunted weight gain in 6–8-week-old, male Swiss Webster mice (Fig. 2B). The alteration of body weight was not due to reduced feeding overall (Fig. 2C). Although there was reduced feeding observed in the Ghr1-KLH animals after the vaccine course had been completed on day 35, this was not specific to the presence of anti-ghrelin antibodies (Fig. 2D). Thus, the alteration in body weight is thus likely to reflect alterations in metabolic processing and energy storage^69,70^.

In contrast to its significant effects on weight, when vaccinated mice were trained to associate cocaine with a specific environment using a conditioned place preference (CPP) assay, no difference was seen between the Ghr1-KLH vaccinated animals and the control groups (Fig. 2E)^69^. Although both mock and active vaccination showed a trend toward reduction of locomotor activity overall, this reduction was not related to selective blockade of the hyperlocomotor effects of cocaine (Supplemental Fig. 1). Therefore, peripheral acyl-ghrelin sequestration may be sufficient to achieve alterations in weight trajectory, whereas blockade of peripheral acyl-ghrelin access to the CNS is not sufficient to alter cocaine-induced rewarding effects. Overall, these results using an active vaccination strategy in cocaine reward are consistent with previous findings from the use of a Speigelmer construct in alcohol reward, providing a generalized basis to indicate that peripheral restriction of acyl-ghrelin is not sufficient to block drug reward in the absence of central alteration of reward processing^72^.

### Acute blockade of GHSR_1a_ blunts weight gain and cocaine-induced place preference

**Figure 3:**
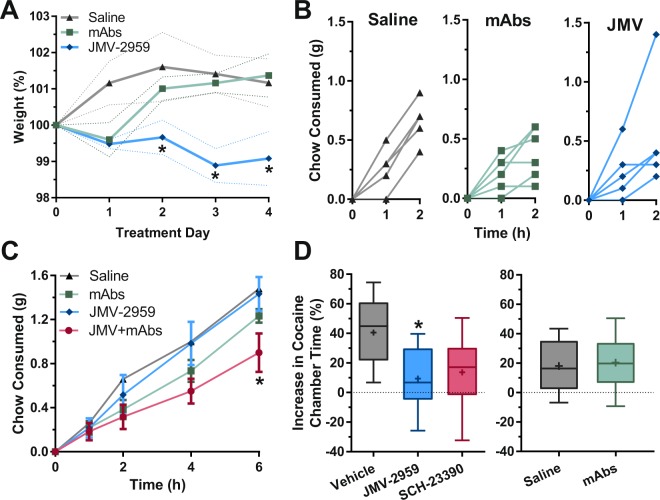
Effect of Acute GHSR_1a_ Antagonism or Peripheral Ghrelin Sequestration on Weight, Feeding, and Reward Processing Behaviors in Mice. (**A**) Normalized body weight over time following treatment with saline (*n* = 5), monoclonal anti-ghrelin antibodies (15 mg/kg daily; *n* = 6), or JMV-2959 (6 mg/kg daily; *n* = 6) [Repeated measures two-way ANOVA (*P*_interaction_ = 0.0304, F_8, 60_ = 2.32; *<0.05 vs Saline, Bonferroni correction)]. (**B**) Chow consumption on day four following repeated treatment with saline (*n* = 5), monoclonal anti-ghrelin antibodies (15 mg/kg; *n* = 6), or JMV-2959 (6 mg/kg; *n* = 6) [Repeated measures two-way ANOVA (*P*_interaction_ = 0.3707, F_4, 28_ = 1.11)]. (**C**) Chow consumption following acute treatment with saline (*n* = 5), JMV-2959 (6 mg/kg; *n* = 6), monoclonal anti-ghrelin antibodies (15 mg/kg; *n* = 6), or both (*n* = 6) [Repeated measures two-way ANOVA (*P*_interaction_ = 0.0127, F_12, 76_ = 2.35; *<0.05 vs JMV-2959, Bonferroni correction)]. (**D**) Conditioned place preference for cocaine (20 mg/kg) in mice treated with saline (*n* = 7), JMV-2959 (6 mg/kg; *n* = 11), or SCH-23390 (1 mg/kg; *n* = 8), [One-way ANOVA [*P* = 0.0286 F_2, 23_ = 4.17, *<0.05 vs Saline, Bonferroni correction)]. Conditioned place preference for cocaine (20 mg/kg) in mice treated with saline (*n* = 8) or monoclonal anti-ghrelin antibodies (15 mg/kg; *n* = 8), (Student’s t-test [*P* = 0.8029]). Data are shown as mean ± SEM (a,c), individual data points (b), or median with quartiles ±5–95% CI; +, mean (d).

To determine the acute effects of ghrelin sequestration and receptor blockade, mice were treated with either JMV-2959, a GHSR_1a_ antagonist, or a combination of three high-affinity monoclonal antibodies (mAb; [JG2: JG3: JG4; 5 mg/kg each]). JMV-2959 blocked weight gain over a four-day treatment period, whereas potent blockade of peripheral ghrelin did not alter body weight trajectory over this time frame (Fig. 3A). Assessment of feeding behavior at the end of this treatment course did not reveal an effect of either treatment in mice that had been fed *ad libitum* (Fig. 3B)^73^. However, when the mAb triplet was combined with JMV-2959 and feeding was assessed over a 6 h period, chow consumption was significantly reduced compared to JMV-2959 alone (Fig. 3C).

Additionally, when JMV-2959 was acutely administered during the test phase of the place preference trial, cocaine-induced CPP was significantly reduced, performing similarly to direct antagonism of dopamine receptor D1 (DRD1) using SCH-23390 (Fig. 3D). Mice treated with the mAb triplet revealed no effect on cocaine reward. JMV-2959 also blunted locomotor behavior on its own (Supplemental Fig. 2). Administration of the putative peripherally-restricted GHSR_1a_ antagonist CCY-2308 was inconclusive, due to confounding effects of the vehicle even when given at minimal volumes (Supplemental Fig. 3). Furthermore, in contrast to previous reports, CCY-2308 appeared to be CNS penetrant (Supplemental Fig. 4)^74,75^. Comparing these results of acute intervention to the effects of vaccination above, these data indicate that blockade of central GHSR_1a_ receptors is sufficient to decrease weight gain and block cocaine rewarding effects. Importantly, these results are consistent with a generalized concept of ghrelin-independent reward blockade through central GHSR_1a_ antagonism, given previous results that demonstrate that JMV-2959 blocked alcohol, opioid, and nicotine rewarding effects^76–78^.

## Discussion

**Figure 4:**
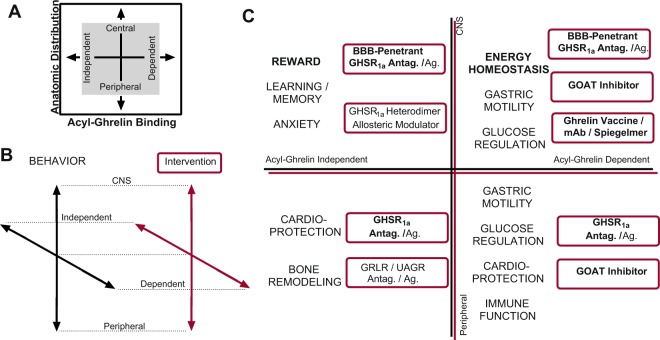
Model for Ghrelin Signaling Regulation and Selective Mechanistic Intervention. (**A**) Two-dimensional model of passive regulation for acyl-ghrelin: GHSR_1a_ signaling. (**B**) Depiction of conceptual overlay for a framework matching behaviors (BLACK) and interventions (Red, boxed) using the two-dimensional model. (**C**) Framework predictions for methods to intervene in the acyl-ghrelin: GHSR_1a_ signaling system more selectively. Empirically observed behaviors under the control of this system (BLACK) are matched with their relevant interventions (Red, boxed) by quadrant.

Overall, these observations agree with previous results suggesting that the effects of ghrelin signaling on body weight gain and food reward can be manipulated independently from one another. Our results further indicate that such independent manipulation is possible because the central GHSR_1a_ that regulate weight gain behavior are physiologically dependent on peripheral acyl-ghrelin activation under normal conditions, whereas hedonic reward due to cocaine administration does not appear to directly require peripherally-synthesized acyl-ghrelin. Together with other available data, this information can be used to develop a preliminary, but testable, two-dimensional model of passive regulation for acyl-ghrelin—GHSR_1a_ signaling functions (Fig. 4)^15,16,53,55,79^. For example, it is known that peripherally-restricted GHSR_1a_ antagonists will lower blood glucose without affecting energy homeostasis or reward, whereas a BBB-penetrant GHSR_1a_ antagonist will alter all three of these outcomes in rodents^80^. Recent work suggests that ligand-independent central GHSR_1a_ blockade can selectively alter reward behavior without inducing substantial adverse metabolic effects in humans^81,82^. Given the evidence for robust GHSR_1a_: DRD_1_ heterodimer expression in the mesolimbic dopaminergic system, we speculate that development of heterodimer-specific allosteric modulators could be an excellent candidate method for further testing this finding.

Within this basic model, the anatomical dimension supports selective activity at peripherally- *versus* centrally-expressed GHSR_1a_, and the differential ligand dependence of GHSR_1a_ sub-populations adds a second acyl-ghrelin-dependent *versus* -independent dimension of selectivity. However, because some complex behaviors are likely to engage multiple functional domains, multi-component interventions may be required to produce robust changes in behavior and physiology. For example, feeding behavior has both a hedonic reward component as well as a homeostatic energy storage component, perhaps necessitating concerted intervention at both the ligand and receptor levels, as suggested by the results in this study and others. This minimal model is presented as a framework for assessing functional utility of prevailing interventional mechanisms; it is not intended as an exhaustive description of the regulatory mechanisms impinging on ghrelin signaling. Because there are likely to be future developments highlighting behaviors where additional regulatory mechanisms, such as desacyl-ghrelin or GHSR_1b_, are involved, it is anticipated that future investigation into these topics will inform expansion, alteration, or rejection of this speculative model^79,83^.

## Methods

### Synthesis of the Ghr1 Hapten

Aminomethyl resin was placed into a fritted 25 mL syringe fitted with a stopcock. The resin was swollen with 20 mL dichloromethane for 20 min. Rink amide, 6-Chloro-1-hydroxybenzotriazole, and N,N′-diisopropylcarbodiimide were dissolved in 10 mL N,N′-dimethylformamide, and nitrogen was bubbled through this mixture for 20 min. The dichloromethane was then drained from the syringe, then the Rink Amide mixture was added to the resin slurry and shaken on a rotary shaker for 90 min. The syringe was then drained, and the resin was washed 4x with 10 mL N,N′-dimethylformamide and 3x with 10 mL dichloromethane. The resin was next treated with 1.1 mL acetic anhydride and 1.1 mL pyridine in 10 mL dichloromethane to cap any unreacted amine residues on the resin, and shaken for 30 min. This mixture was then drained, and the resin was washed as above. The syringe was then filled with 25% piperidine in N, N′-dimethylformamide (10 mL) to cleave the Fmoc group from the rink amide, and shaken for 30 min. The syringe was once again drained and washed as above. This process of couple, wash, deprotect, wash, was repeated sequentially with amino acids proceeding from the C-terminus of the hapten (C,Q,H,E,P,S,L,F,S,S,G). Each coupling was allowed to proceed for 90 min, and each deprotection was allowed to proceed for 30 min (two 15 min portions, with replacement of the reagents in between). Progression of the reaction was monitored using the Kaiser test. The final amino acid (glycine, G) was added as a Boc protected version to allow for global deprotection following Ser^3^-butanoylation. The resin was treated with 0.1 M tetrabutylammonium fluoride in N,N′-dimethylformamide for 30 min (2 × 15 min) to selectively deprotect the TBDMS group of Ser^3^. The resin was then washed with N,N′-dimethylformamide (3 × 10 mL) and NMP (3 × 10 mL), and treated with butyric anhydride for 4 h, and catalytic 4-dimethylaminopyridine in N-methylpyrollidinone for 12 h to butanoylate Ser^3^. The resin was then washed with 10 mL N, N′-dimethylformamide (4x) and dichloromethane (3x), methanol (2x), diethyl ether (2x), and dried under vacuum for 2 h. The resin was then placed in a round bottom flask and cleaved off the solid support with 95% trifluoroacetic acid, 2.5% H_2_O, and 2.5% triisopropylsilane (15 mL) for 3 h. After filtration, the cleaved product was isolated by adding the peptide trifluoroacetic acid solution dropwise to chilled diethyl ether: petroleum ether (3:1–4 × 10 mL), giving a white precipitate. This was centrifuged for 10 min at 6500 rpm and decanted. The solid was washed with diethyl ether, centrifuged, and decanted four times, then dried under vacuum. Final purification was accomplished by preparative HPLC on C_18_ using 10–60% acetonitrile [0.1% trifluoroacetic acid] in H_2_O [0.1% trifluoroacetic acid]). Confirmation of the correct mass was accomplished using ESI Mass Spec (C_18_ column − 214 nM; 10–90% acetonitrile [0.1%trifluoroacetic acid] in H_2_O [0.1% trifluoroacetic acid]; m/z = 1260.9821).

### Conjugation of the Ghr1 hapten

Sulfo-GMBS (N-γ-maleimidobutyryl-oxysuccinimide ester) was dissolved in 0.9 mL phosphate buffered saline (PBS), then 0.1 mL of Keyhole limpet Hemocyanin (KLH) (10 mg/mL in PBS) was added. This reaction was then allowed to stir for 16 h at 4 °C. Ghr1 was then added to the reaction mixture, and the reaction was allowed to stir for 8 h at room temperature. The reaction mixture was then dialyzed against PBS using a Zeba spin column (10 mL; 7 K MWCO) to obtain the purified conjugate. A corresponding sample conjugated with BSA was run using the same conditions, and MALDI was used to verify the conjugation occurred. Copy number was calculated as (Ghr1-BSA Mass − BSA Mass)/(Ghr1 Mass − Water Mass).

### Antibody binding

Microtiter plates (Costar 3690) were incubated with coating antigen Ghr1-BSA in PBS (5 µg/mL, 25 µL; 18 h, 37 °C). 5% non-fat milk in PBS (30 min, 37 °C) was added to block non-specific binding. Mouse sera in 1% BSA were serially diluted across the plate before incubation in a moist chamber (1.5 h, 37 °C). The plate was washed with deionized H_2_O before incubation with peroxidase-conjugated donkey anti-mouse IgG (Jackson ImmunoResearch Laboratories, Inc.; Catalog # 715-035-151) in a moist chamber (30 min, 37 °C). The plates were further washed with deionized H_2_O before being developed with the TMB substrate kit (Thermo Pierce) and the absorbance at 450 nm measured on a microplate reader (SpectraMax M2e Molecular Devices). Titers were calculated as the dilution corresponding to 50% of the maximum absorbance from a plot of the absorbance versus log (dilution). Surface plasmon resonance (SPR) binding analysis was conducted on a Biacore 3000 instrument (GE Healthcare) equipped with a research-grade CM5 sensor chip. BSA and the hapten-BSA conjugates were immobilized in two different flow cells using NHS/EDC coupling chemistry and dilution of anti-sera was performed to normalize baseline binding across samples. Serum was pre-incubated with compound at room temperature for 30 min, the mixture was injected over both flow cells for 5 min and dissociated for 2.5 min in running buffer (HBS-EP + buffer, pH 7.4, GE Healthcare) before the surface was regenerated with Gly-HCl at pH 1.5.

### Vaccine preparation

The active Ghr1-KLH-Alum-CpG ODN 1826 vaccine formulation for injection was composed of a 1:1:1 (v/v/v) mixture of Ghr1-KLH immunoconjugate (1 mg/mL in PBS), CpG ODN 1826 (1 mg/mL in PBS), and Imject Alum (3 mg/mL in PBS). For each active injection, one aliquot each of Ghr1-BSA, CpG ODN 1826, and Alum were diluted to the correct concentrations, then mixed together to form the vaccine formulation, and shaken at room temperature for 30 min. After shaking, the vaccine was stored on ice for no more than 1 h prior to injection. The control KLH-Alum-CpG ODN 1826 vaccine formulation for injection was generated through the same procedure, only starting with a 1:1:1 (v/v/v) mixture of KLH (1 mg/mL in PBS), CpG ODN 1826 (1 mg/mL in PBS), and Imject Alum (3 mg/mL in PBS).

### Animals

Animal studies were approved by TSRI’s Institutional Care and Use Committee and carried out according to NIH guidelines. Age-matched male Swiss Webster mice (6–8 weeks or 12–14 weeks) were acquired for the study (Taconic Biosciences). The animals were housed 4 per cage in a temperature-controlled (22 °C) vivarium in ventilated cages with cedar bedding, on a reverse light cycle (dark 9 AM–9 PM; light 9 PM–9 AM), allowed *ad libitum* access to food (Envigo, 2018 Teklad Global 18% protein diet) and water (except where otherwise noted) with a polyvinylchloride tube and nesting materials provided for enrichment. The cages were changed by vivarium staff once per week.

### Drugs

JMV-2959 and SCH-23390 (>95%, Tocris Biosciences) were obtained commercially and used without further purification. CCY-2308 (>95%; m/z = 550.2526) was synthesized and purified at TSRI using the published method^75^. For *in vivo* studies, drugs were given intraperitonally (IP) at a volume of 10 mL/kg in bacteriostatic saline (0.9% w/v), aside from CCY-2038, which was given at a volume of 2 mL/kg, using a 80:20 Polyethylene glycol 400: Methanesulfonic acid (10 mM in 0.9% bacteriostatic saline) vehicle, as previously described^75^.

### Vaccination Procedure

The animals were habituated to the vivarium facility for 1 week prior to initiation of study, then each group of littermates was stratified into treatment groups to provide an equivalent baseline weight for each group. Each animal was injected IP with 150 µL of vaccine formulation, delivering a dose of 50 µg Ghr1-KLH, 50 µg CpG ODN 1826, and 150 µg of Alum per animal for each injection. The animals were injected with a priming dose on day 0, with boosts provided on days 14 and 28. Retro-orbital bleeds were taken on days 21 and 35 under light isoflurane anesthesia to assess the antibody titers generated for each animal. Animals underwent weight assessment, feeding assessment, and conditioned place preference during this time period, as outlined below.

### Weight assessment

Animals were weighed in the middle of the dark cycle (1 PM–2 PM) using a balance with a sensitivity of ±0.1 g. Weight gain or loss was normalized to initial body weight using the formula: Normalized Weight = (Current Weight/Baseline Weight) × 100.

### Feeding assessment

Feeding was assessed in the middle of the dark cycle (1 PM–2 PM) using a balance with a sensitivity of ±0.1 g. Animals were transferred to clean individual cages with bedding and provided with access to 18–21 g of their standard chow. The individual pellets were weighed at 1 and 2 h (and 4 and 6 h where shown) and the amount of food consumed was quantified. Animals did not have access to water or any enrichment materials during the 2 h (or 6 h) observation period. In vaccinated animals, feeding was assessed on days −3, 3, 17, and 31. For acutely treated animals, feeding was assessed immediately after injection on either day 1, or on day 4 for those animals with multiple days of treatment.

### Conditioned place preference

For the conditioned place preference test, vaccinated animals were tested on days 36–42 in a dedicated 4.6 × 4.6 m room lit by a 30 W upward-directed light source in the center of the room, with the brightness across all areas of each apparatus measured at 45–50 lux. All animals were placed in a clean plastic box apparatus (267 × 483 × 203 mm) divided by color (black vs white walls) and texture (smooth vs textured floor) and tracked for 20 min (1 PM) for assessment of baseline preference. Subsequently, a wall was placed in between the two chambers during the conditioning phase. Animals received saline and were placed in their more-preferred chamber for 20 min (8 AM). Later, animals received 20 mg/kg cocaine and were placed in the less-preferred chamber for 20 min (6 PM). This conditioning procedure was repeated for three days. On the test day, the dividing wall was removed, the animals were treated with saline or GHR_1a_ antagonist 30 min prior to testing, and the animals were tracked for 20 min (1 PM). The preference for the drug-paired chamber before and after training was calculated using the formula: Preference = (chamber time post training– chamber time pre-training). The experimenter was blinded to group identity during data processing, and behavioral results were automatically scored using AnyMAZE v.4.99 (Stoelting Co). Any animal that did not fully explore the apparatus during either the baseline or test phase, using the criterion of spending ≥85% of time in a single chamber, was removed from the study.

### Locomotor activity

Mice were injected with either saline or 20 mg/kg cocaine before being placed in a plastic cage (267 × 483 × 203 mm) with clear ventilated acrylic top to be recorded and tracked by overhead camera using ANY-Maze video tracking software (Stoelting Co., Wood Dale, IL). Sessions were run for 20 min during the middle of the dark cycle (1 PM) in a 4.6 × 4.6 m room with a single 30 W upward-directed light source and repeated on mice after a two-day washout period until all mice had been assessed three times under both conditions. The average of the three assays was taken for each condition. The percent increase in locomotion exhibited in the presence of cocaine was calculated using the formula: % Increase = (Cocaine distance/Saline distance) × 100.

### LC/MS Analysis of CCY-2308

6–8-week-old, Male Swiss Webster mice (Taconic Biosciences) were injected IP with the test compound, CCY-2308. Blood was taken via the retro-orbital route; brain tissue was immediately flash-frozen using acetone: CO2(s). Tissues were stored at −80 °C until analysis. For serum samples, 20 μL were added to 80 μL of methanol +200 ng internal standard. This was centrifuged for 10 min at 10,000 r.p.m., and 60 μL were transferred to a LC–MS vial. Brain homogenate was diluted four times with 7:1 (vol/vol) methanol/water. After centrifugation, 1 mL of this sample was collected and 200 ng of internal standard (fenethylline-d^3^) was added. The mixture was stirred for 2 h, centrifuged, solvent collected, dried under vacuum and then re-suspended in 60 μL methanol. Analysis was carried out on an Agilent 300SB-C8, 4.6 × 50 mm. LC–MS system with a Poroshell 300 SB-C8 column using H2O/ACN (with 0.1% formic acid) as the mobile phase (is T0 = 95:5, T6 = 5:95, T10 = off; 3.5 min re-equilibration time). To enhance the accuracy and precision of the mass of interest, a selective ion monitoring (SIM) method was used. For the internal standard fenethylline-d^3^, the mass range of 345.1–345.5 was extracted, and for CCY-2308, mass range of 549.8–550.2 was extracted for integration. Using the ratio of drug to internal standard integration values, the unknown serum and tissue concentrations were fit to a standard curve.

### Statistical analysis

Sample sizes were calculated to give >80% power using means and standard deviations from our previous results for the ghrelin vaccines as well as literature results for the small molecules. Where ‘*n*=’ is listed, it represents the number of animals used for analysis. Data were graphed and analyzed using Prism 5.02 (GraphPad Software), setting *P* < 0.05 as the critical value. Groups were analyzed for similarity of variance using Bartlett’s Test, and non-parametric tests were employed where the data was found to be non-normally distributed. All statistical tests were performed using two-tailed analysis.

## Supplementary information

Supplemental Information

## Data Availability

All data supporting these findings will be provided upon request.
