# Supplementary material for: Ghrelin Receptor Influence on Cocaine Reward is Not Directly Dependent on Peripheral Acyl-Ghrelin
Source: Sci Rep. 2019 Feb 12;9:1841. doi: 10.1038/s41598-019-38549-z (PMC6372697; doi:10.1038/s41598-019-38549-z)
Supplement: Supplementary file 1 — Supplemental Information [file 41598_2019_38549_MOESM1_ESM.pdf]

SUPPLEMENTAL INFORMATION

TITLE

Ghrelin Receptor Influence on Cocaine Reward is Not Directly Dependent on Peripheral Acyl-Ghrelin

AUTHORS

Cody J Wenthur, Ritika Gautam, Bin Zhou, Leandro F Vendruscolo, Lorenzo Leggio, Kim D Janda

Supplemental Table 1. Ghr1 Copy Number Following Bioconjugation to Bovine Serum Albumin

**Species**	**Replicate**	**Mass**	**Diff. from BSA**	**Copy #**
BSA		*66463*		
Ghr1-BSA	1	72579	6116	5
Ghr1-BSA	2	83159	16696	13
Ghr1-BSA	3	79085	12622	10

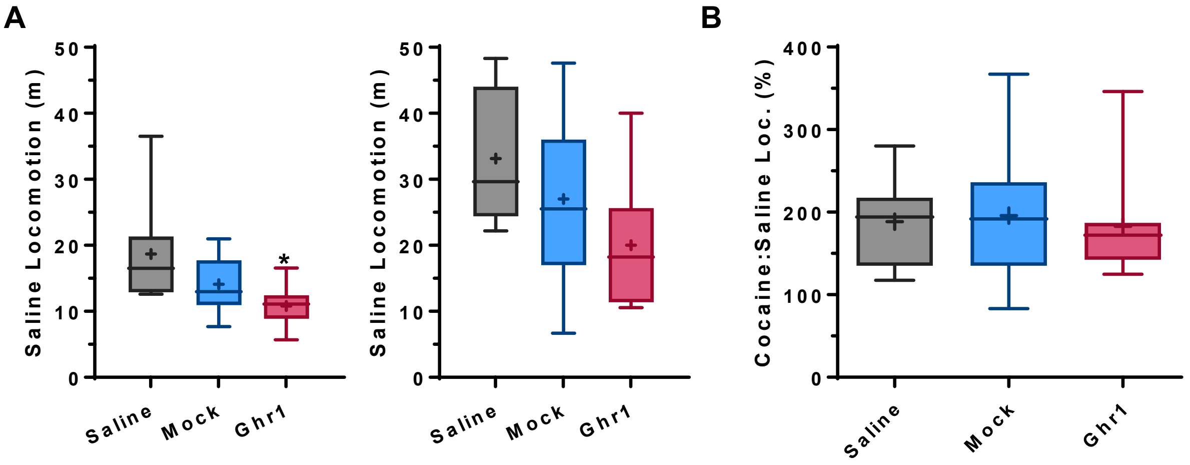

Supplemental Figure 1. Locomotor effects of vaccination against ghrelin. A) Locomotor activity after treatment with saline [One-way ANOVA (P = 0.0173, $F_{2, 27}$ = 4.73, * p<0.05 *vs.* Saline, Bonferroni correction)] or cocaine (20 mg/kg; [One-way ANOVA (P = 0.0547, $F_{2, 27}$ = 3.24)]) in the presence of saline pretreatment (n = 8), vaccination with KLH (n = 9), or Ghr1-KLH (n = 9). B) Percent increase in cocaine locomotion compared to saline baseline [One-way ANOVA (P = 0.9145, $F_{2, 27}$ = 0.09)]. Data are shown as median with quartiles ± 5-95% CI; +, mean.

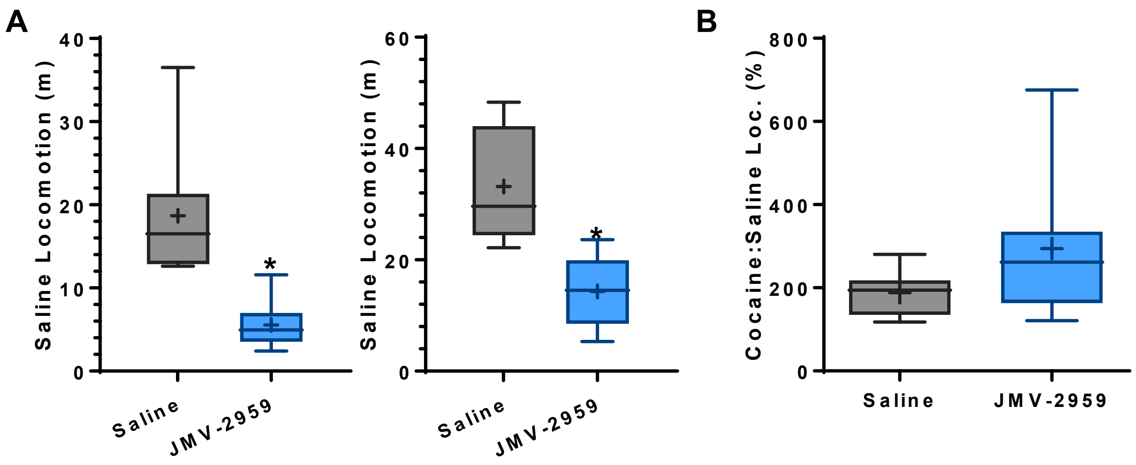

Supplemental Figure 2. Locomotor effects of JMV-2959. A) A) Locomotor activity after treatment with saline [Student's t-test (*P* = 0.0007, * p<0.05 *vs.* Saline)] or cocaine (20 mg/kg; [Student's t-test (*P* = 0.0005, * p<0.05 *vs.* Saline)] in the presence of saline pretreatment (*n* = 8), vaccination with KLH (*n* = 9), or Ghr1-KLH (*n* = 9). B) Percent increase in cocaine locomotion compared to saline baseline [One-way ANOVA (*P* = 0.9145, $F_{2, 27}$ = 0.09)]. Data are shown as median with quartiles ± 5-95% CI; +, mean.

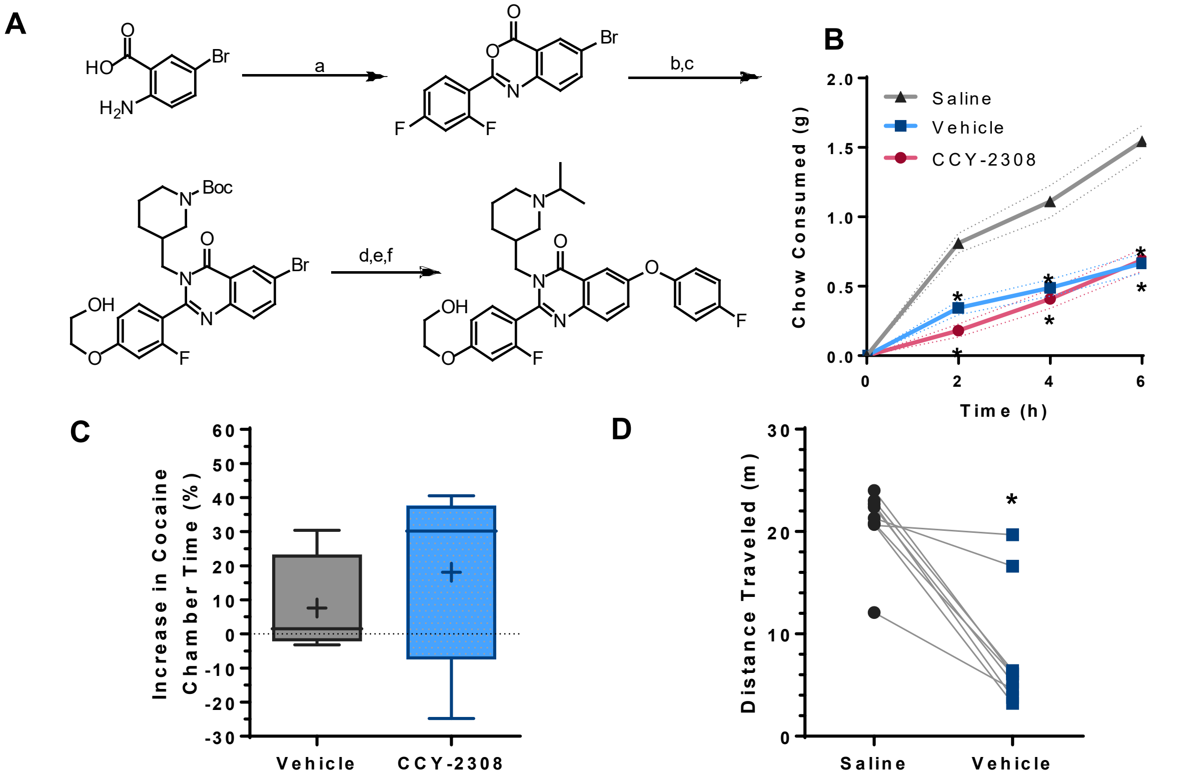

Supplemental Figure 3. Effect of CCY-2308-mediated $GHSR_{1a}$ antagonism on feeding and cocaine reward in mice. A) Synthetic route to access CCY-2308. Conditions: a-2,4-difluorobenzoyl chloride, Et_3N, DCM, 23 °C, 2 h AcOH, 50 °C, 1.5 h (yield: 97%); b – Boc-(3-aminomethyl)piperidine, toluene, 130 °C, 4 h. c – Ethylene glycol, K_2CO_3, 140 °C, 8 h (yield: 11%). d-4-fluorophenol, Cs_2CO_3, Cu(I)Cl, dimethylglycine, NMP, 115 °C, 10 h. e – TFA, DCM, 23 °C, 4 h. f – 2-Iodopropane, K_2CO_3, ACN + 1% H_2O, 80 °C, 10 h (yield: 29%). B) Chow consumption following acute treatment with saline (n = 5), vehicle (80:20 PEG 400:10 mM $MeSO_4$ in saline; n = 6), or CCY-2308 (30 mg/kg; n = 6) [Repeated measures two-way ANOVA ($P_{interaction} < 0.0001$, F6, 75 = 21.93; *p <0.05 vs Saline, Bonferroni correction)]. C) Conditioned place preference for cocaine (20 mg/kg) in mice treated with vehicle (80:20 PEG 400:10 mM $MeSO_4$ in Saline; n = 4), or CCY-2308 (30 mg/kg; n = 5), [Student's t-test, (P=0.5080)]. Nine animals were removed from the study due to failure to meet the threshold of < 85 % of time in one chamber for exploration of the apparatus. D) Locomotor activity measured in the presence of saline (n = 9) or vehicle (n = 9) [Student's t-test (P =0.004, *p<0.05 *vs*. saline)]. Data shown as mean ± SEM (b), median with quartiles ± 5-95% CI; +, mean (c), or individual data points (d).

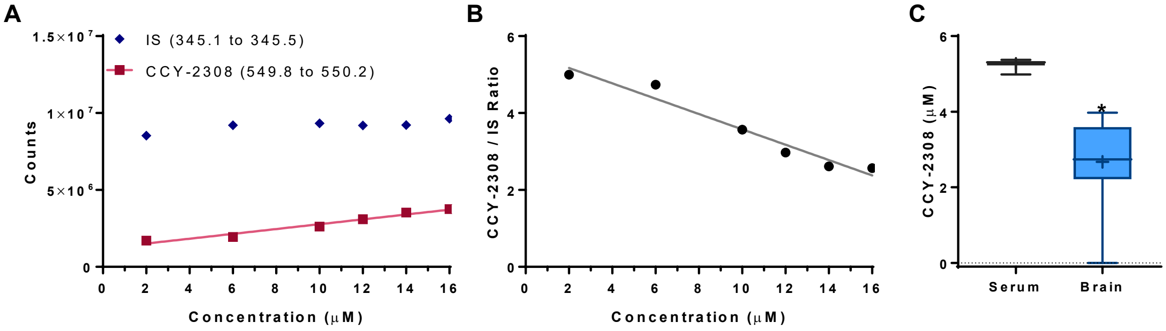

Supplemental Figure 4. Quantification of CCY-2308 in serum and blood. A) Mass spectrometric quantification of CCY-2308 and internal standard (IS) to generate standard curve. B) Ratios of CCY-2308/IS used to interpolate values onto standard curve. C) Serum (*n* = 10) and brain (*n* = 10) concentrations of CCY-2308. (Paired Student's t-test [$P < 0.0001$]).
